# Renewable saccharide-derived porous carbon foams with metal particles for CNT and graphene substrates in electrochemical applications

**DOI:** 10.1016/j.isci.2025.112050

**Published:** 2025-02-17

**Authors:** Yi Ma, William Coley, Ruoxu Shang, Fabian Villalobos, Pedro Pena, Andrew Patalano, Evan Jauregui, Nicholas Roskopf, Mihrimah Ozkan, Cengiz S. Ozkan

**Affiliations:** 1 Department of Mechanical Engineering, University of California, Riverside, Riverside, CA 92521 USA; 2 Materials Science and Engineering Program, University of California, Riverside, Riverside, CA 92521 USA; 3 Department of Chemistry, University of California, Riverside, Riverside, CA 92521 USA; 4 Department of Electrical and Computer Engineering, University of California, Riverside, Riverside, CA 92521 USA; 5 Department of Mechanical Engineering, University of California, Riverside, Riverside, CA 92521 USA

**Keywords:** Materials science, Materials chemistry

## Abstract

Harnessing the potential of renewable saccharides, porous carbon foams embedded with metal nanoparticles have been innovatively synthesized, pushing the boundaries of electrochemical applications. By embedding metal nanoparticles in carbon foams derived from saccharides and metal salts, substrates for the growth of multi-walled carbon nanotubes (MWCNTs) and multi-layer graphene through chemical vapor deposition (CVD) are created. The influence of varying saccharide-to-metal precursor ratios on the morphology of these nano-structured carbons is examined. The diameters of carbon nanotubes formed at different saccharide to nickel (Ni) ratios are compared, with corroborative insights from X-ray diffraction (XRD) and Raman spectroscopy. Substituting cobalt (Co) salts for Ni precursors reveals notable differences in carbon morphologies. The resulting carbon nanotube-carbon foam composites exhibit remarkable properties, enabling the creation of hierarchical carbon foams. Furthermore, their potential as carbon electrodes for electrochemical double-layer capacitors (EDLCs) is evaluated, highlighting their promise in cutting-edge electrochemical applications.

## Highlights

• Porous carbon foam with metal nanoparticles enables nanocarbon growth in MWCNTs and graphene
• The saccharide-to-metal ratio significantly impacts the nano-structured carbon’s morphology
• Carbon nanotube-foam composites are superhydrophobic and oleophilic, ideal for oil recovery
• As active electrode materials, supercapacitors reached 73.45 F/g (K^+^) and cycled stably

## Graphical abstract

**Figure:**
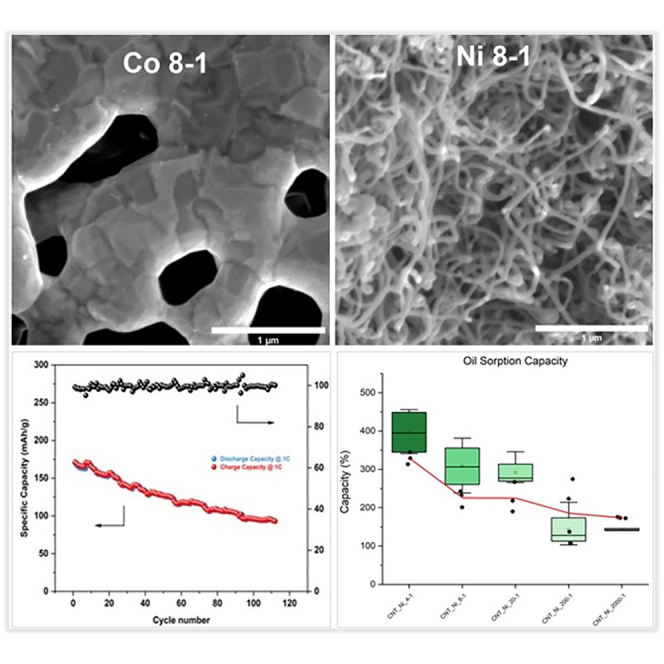

## Introduction

Porous carbon materials (PCMs) have emerged as a versatile and crucial class of materials in energy storage, environmental remediation, catalysis, and biomedical applications. Their high surface area, tunable pore structure, and excellent conductivity make them attractive candidates for capacitors, batteries, adsorbents, and photocatalysts. Advances in fabrication techniques and structural modification strategies are expanding the potential applications of PCMs. The synthesis of PCMs primarily involves methods such as template-based approaches, activation techniques, and self-assembly.^1^^,^^2^

The applications and characteristics of porous carbons are well-documented in scientific literature. These applications can encompass filtration, fluid absorption and storage, and serve as conductive additives in electrochemical energy storage,^3^^,^^4^^,^^5^ among others. Despite refinements and expansions in their use, these carbon materials’ fundamental nature has remained unchanged in recent years. Activation processes, used to create micro- and meso-pores, are commonly applied to carbons with a macrostructure or those derived from natural materials such as coconut shells, wood, and other cellulosic materials.^5^^,^^6^^,^^7^^,^^8^^,^^9^^,^^10^ These chemical and physical activation methods increase surface area and control porosity. High-temperature treatments are also utilized to enhance graphitization and, consequently, conductivity in glassy carbons or, conversely, in diamond-like carbons.^11^^,^^12^^,^^13^^,^^14^^,^^15^^,^^16^^,^^17^ However, sufficient focus has not been given to functionalizing these PCMs through other means for different properties. Carbon foams offer a unique pathway toward innovative applications. These foams, characterized by their large macro-structure, can serve as a scaffold for developing new composite materials through post-treatments like chemical vapor deposition (CVD). By infusing these carbon foams with metal nanoparticles, researchers have the potential to create porous carbon matrices with catalytic particles embedded within their macrostructure.^18^ These metal nanoparticles provide a means to incorporate additional properties into the overarching carbon matrix. Iron (Fe), nickel (Ni), and cobalt (Co), which are magnetic and maintain their ferromagnetic properties at the nanoscale, can be embedded in a carbon matrix.^19^ The low density of the carbon does not impede the magnetic attraction experienced by the metal particles when exposed to a magnetic field. As a result, magnetic properties can be indirectly incorporated into porous carbon. Furthermore, the metal particles offer potential chemical mechanisms and phase separations that can be exploited. Ammonia catalysts heavily depend on magnetite and Fe. A porous catalyst could be cultivated from a solution by creating scaffolds with embedded Fe nanoparticles. Altering the metals changes the chemical reactions that can be utilized. These same metals can serve as substrates for the growth of nanostructures such as nanotubes,^16^^,^^19^^,^^20^^,^^21^ 2D sheets,^22^ and encapsulations.^23^^,^^24^

**Figure 1:**
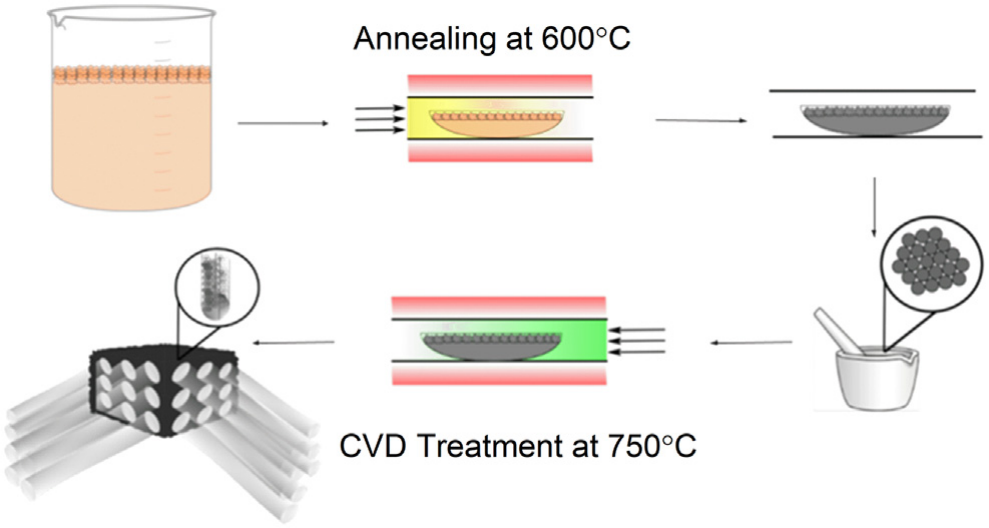
Synthesis schematic Top left: Mixing and foaming step, Top Middle: annealing for particle growth step, Top Right: annealed particles embedded within carbon foam, Bottom Right: milling by mortar and pestle and sieving to control carbon foam chunk size, Bottom Middle: CVD treatment for CNT and Graphene growth, Bottom Left: final growth of CNTs or graphene.

This work introduces a feasible and low-cost technique for cultivating carbon nanotubes and graphene sheets on carbon foams using embedded metal particles, as shown in Figure 1, expanding their potential uses in oil absorption (Table 1) and electrochemical energy storage. Carbon foams are created from basic polysaccharides and metal salts to establish a porous, large-scale macrostructure and are then annealed to initiate metal nanoparticles. An investigation is carried out to identify the potential shapes of nano-structured carbons when metals with varying carbon solubility are used as substrates for phase separation at different ratios of sugar to metal precursors (Table 2). By altering the carbon-to-metal content of the precursors, it becomes feasible to examine the impact of metal grain sizes on the morphology of nano-structured carbons produced by CVD.

**Table 1:** Oil adsorption capacity of as obtained carbon materials compared to other carbon materials

Carbon Material	Oil Adsorption Capacity (w:w %)	Reference
Activated carbon	85–95	Sabzehmeidani et al.^25^
Carbon nanotubes	95–98	Sankaranarayanan et al.^26^
Graphene/Graphene oxide	90–99	Zango et al.^27^
Biochar	75–85	He et al.^28^
Carbon aerogels	99	Nguyen et al.^29^
Graphene-PDMS sponge	20–99	Liu et al.^30^
Graphene-biochar composite	95–98	Liu et al.^31^
Carbon nanofiber	62.6–138.4	De et al.^32^
This work	150–400	–

**Table 2:** Samples prepared in this study and corresponding tests

Sample	Metal Seed for CNT growth	Sugar-to-metal molar ratio
CNT@pC-4	Co, Ni	4:1
CNT@pC-8	Co, Ni	8:1
CNT@pC-20	Co, Ni	20:1
CNT@pC-200	Co, Ni	200:1
CNT@pC-2000	Co, Ni	2000:1

## Results and discussion

### Material characterization

Due to the differences in carbon solubility, different-sized metal particles are expected to nucleate during the 600°C annealing step. This should lead to differences in CNT diameter and, as observed, different morphologies of carbon materials are synthesized when switching from Ni to Co, even at the same ratio. Methods used to characterize these carbon morphologies include SEM and ImageJ software to measure the CNT diameters, SAS statistical software to calculate datasets and normality, and traditional methods like XRD and Raman. Lorentzian peak fitting is used to analyze the Raman spectra to discern the electronic structure and the XRD spectra to calculate the Ni and Co grain sizes used during CVD treatment.

**Figure 2:**
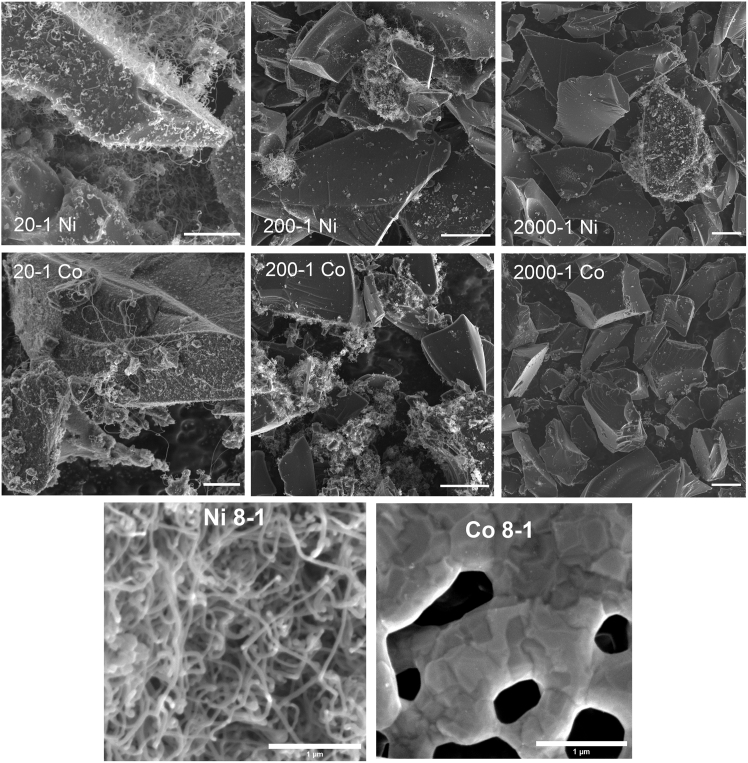
SEM images for CNT growth on Ni- and Co-particles CNT@pC samples with sugar to metal ratio of 20-1 is at 5 μm, 200-1 is at 10 μm, and 2000-1 is at 10 μm. Zoom-in images of the 8-1 samples can be found at the bottom, indicating distinctive morphologies of CVD carbon deposition (All scale bars represent 1 μm).

A comparison of 3 sugar-metal ratios (20-1, 200-1, 2000-1) between Ni and Co is presented in Figure 2. Comparing the 20-1 ratios shows that Ni and Co-produced nanoparticles are available for CNT growth at their surface. However, Ni promotes the growth of longer CNTs than Co. In the Co 20-1 sample, shorter CNTs are more common than longer ones, although CNT diameter does not change much between the ratios. As the xylose content increases to the 200-1 and 2000-1 ratios, Ni and Co particles become sparser, found in small, dense agglomerations where the particles nucleate CNT growth during CVD synthesis. This is demonstrated in Figure 2. At the 8-1 ratio, CNTs successfully coat the Ni-carbon foam. At the 4-1 ratio, CNTs are less prevalent than at 8-1 while also measuring larger in diameter on average. The 4-1 and 8-1 Co-carbon foam did not produce significant CNTs when undergoing CVD—instead, small pseudo-graphitic crystallites formed on the surface of the porous foam, as seen in later sections. Differences in carbon solubility may be responsible for these contrasting morphologies.

**Figure 3:**
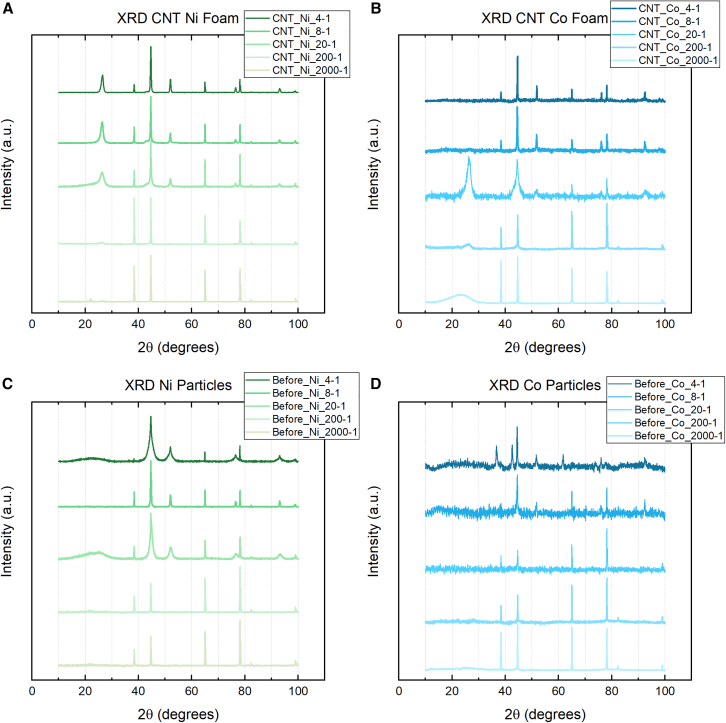
XRD spectra for sugar-metal ratios 4-1, 8-1, 20-1, 200-1, and 2000-1 are depicted (A) CNTs grown on Ni carbon foam substrate. (B) CNTs grown on Co carbon foam substrate. (C) Ni-carbon foam pieces treated at 600°C for 1h. (D) Co-carbon foam pieces treated at 600°C for 1h.

The XRD spectra in Figure 3 indicates the crystallinity of the metal nanoparticles embedded in carbon foam chunks sieved through a 20 μm mesh to undergo CVD treatment (a and b) and pretreatment annealing to 600 C (c and d). Figure 3A indicates the XRD spectra of CNT@pC-Ni with sugar-metal ratio from 4:1 to 2000:1, while Figure 3B shows that of CNT@pC-Co with same ratios. Due to exposure to air, metal oxides are detected in all ratios. For instance, the (311), (400), (440), and (533) reflections are assigned to Co_2_O_3_ of spectra in Figure 3B.^33^ However, at higher metal ratios (4-1, 8-1, and 20-1), the (111), (200), (220), and (311) reflections for FCC Ni and Co are detected, as shown in Figures 3A and 3B, respectively. The (222) reflection is also detected for Ni, as seen in Figure 3A. Grain size diameter for Ni was calculated using full-width half max (FWHM) and Scherrer’s equation for each sugar-metal ratio. Grain sizes were calculated to be 53.51 nm, 50.91 nm, 39.03 nm, 27.99 nm, and 30.03 nm for the 4-1, 8-1, 20-1, 200-1, and 2000-1 ratios, respectively. Grain sizes for Co 2000-1 and 200-1 could not be calculated due to the Absence of significant intensity of Co peaks. FWHM peaks for Co_2_O_3_ cannot be used because CNT grows from pure Co nanoparticles. The process of carbon phase separation during CNT growth encapsulates catalytic Co particles, preserving their crystallinity. Therefore, any oxides present would not have taken part in the growth of CNTs and should not be used to estimate catalytic particle size diameters. Peak analysis and fitting estimated the diameter of Co grains in 20-1, 8-1, and 4-1 ratios of 25.62 nm, 81.48 nm, and 54.99 nm, respectively.

Due to the multi-walled nanotube’s concentric graphene sheets, the XRD spectra strongly resemble those of graphite. The (002) diffraction peak at 27 for graphite is produced at the 4-1, 8-1, 20-1, and 200-1 ratios in the CNT Ni-carbon foam (Figure 3A). The (101) graphite peak may be present at the shoulder of the (111) Ni peak at approximately 44° in the 4-1, 8-1, and 20-1 ratios. Graphite is prominent in the CNT Co-carbon foam at the 20-1 and 200-1 ratios (Figure 3B). Specifically, at the 20-1 ratio, a broad (002) graphite peak is present with a broad Co (111) peak, suggesting that as the carbon nanotubes’ graphitization increases, the Co nanoparticles’ crystallinity decreases. This may be due to differences in carbon solubility at the nanoparticle scale. At the 2000-1 ratio, amorphous carbon (a-C) is likely detected for the CNT Co-carbon foam.

**Figure 4:**
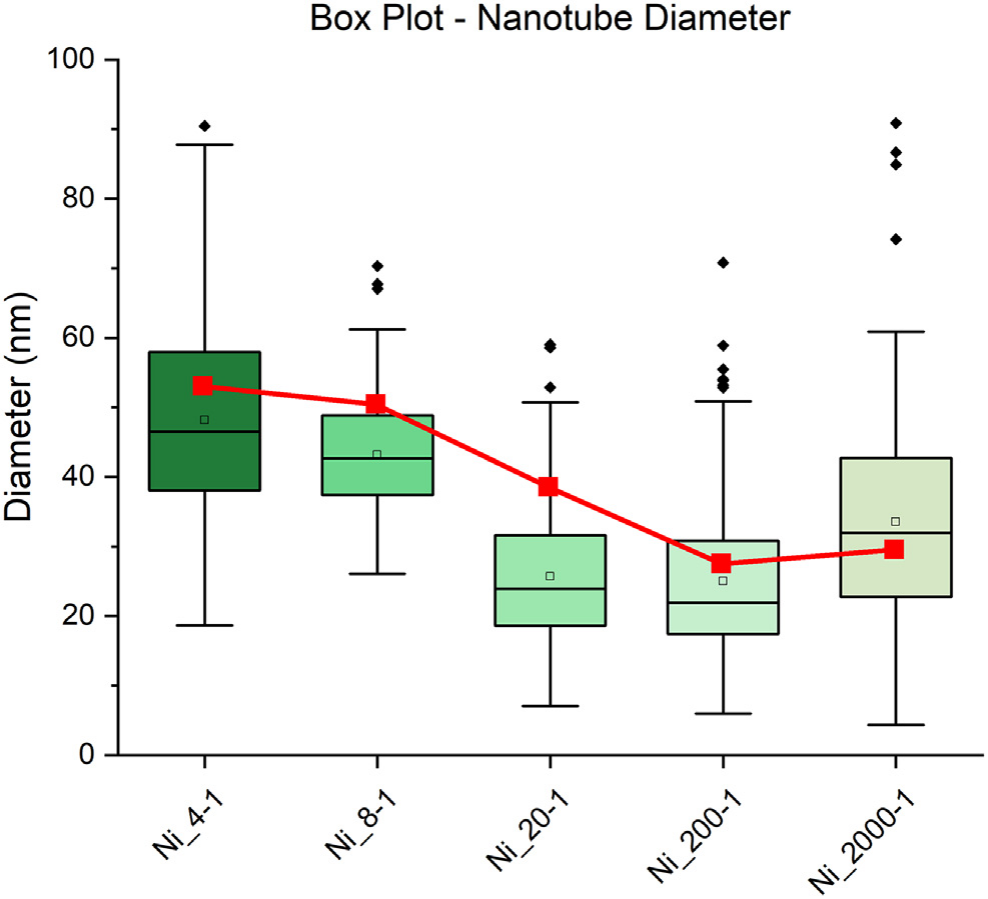
A compilation of the average MWCNT diameter as measured by ImageJ The red line indicates the Ni grain size diameter calculated by Scherrer’s Equation using FWHM of XRD Spectra in Figure 3.

The diameters of CNTs are observed to be dependent on particle diameter, as is commonly noted in the literature^34^^,^^35^ A study of CNT diameter was undertaken to compare the diameters of CNTs between Ni and Co and between the sugar-metal ratios chosen in this study. Approximately 100–200 CNT diameters were measured for each Ni and Co foam that produced CNTs after CVD synthesis. The 4-1 and 8-1 Co-carbon foams exposed to CVD synthesis did not produce a significant number of CNTs and did not produce a large enough sample size to measure. Normality was tested using a Proc GLM test. If distribution was not normal, a Satterthwaite fitting was used. Comparisons between Ni and Co and ratios were done using a Proc TTest to test for significance. Average CNT diameters from Ni for the 4-1, 8-1, 20-1, 200-1, and 2000-1 were calculated to be: 48.13nm, 43.19nm, 25.68nm, 24.99nm, and 33.54nm, respectively. Average CNT diameters from Co for the 20-1, 200-1, and 2000-1 ratios were calculated to be 32.64nm, 33.83nm, and 32.37nm, respectively. A summary of the histograms collected from the data is presented in Figure 4. The average diameters of the Ni CNTs calculated by SAS agree with the diameters of the Ni grain size calculated by Scherrer’s Eq. using FWHM from Figure 3.

**Figure 5:**
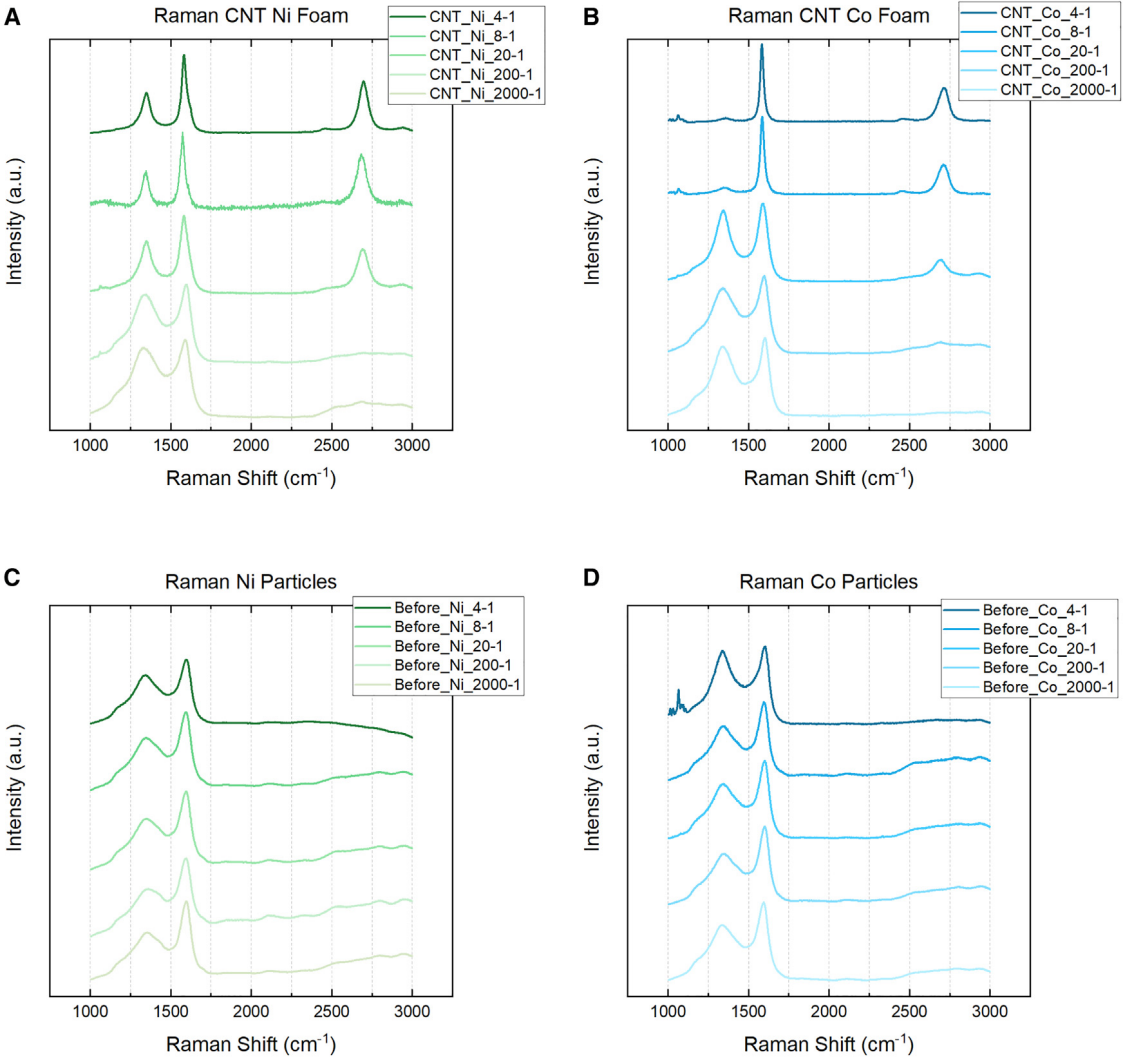
Raman spectra of Ni and Co samples (A) CVD-treated Ni-carbon foam, (B) CVD-treated Co-carbon foam, (C) Ni-carbon foam post-annealing and before CVD treatment, (D) Co-carbon foam post-annealing and before CVD treatment.

Raman spectra were taken for each ratio of the CNT@pC-Ni and CNT@pC-Co samples, as seen in Figure 5. Samples exposed to CVD treatment are shown in Figures 5A and 5B, while samples annealed to 600°C are shown in c and d. All samples annealed at 600°C display a similar spectrum analogous to a-C. In conjunction with the XRD data in Figure 3, the Raman spectra suggest that the nucleation of crystalline nanoparticles without graphitization of carbon is achieved. The 4-1, 8-1, and 20-1 samples that underwent CVD treatment show characteristic graphitic peaks in the Raman spectra. Spectra for the 200-1 and 2000-1 ratios for both Ni and Co also show an amorphous shape that may be due to hydrogenation. Lorentzian peaking fitting was used to demonstrate the appearance of u 1 and u 3 peaks assigned to *trans*-polyacetylene in the 1100-1250cm^−1^ and 1400-1550cm^−1^ ranges, respectively. The detection of a-C is expected as the SEM micrographs in Figure 2 attest to the increasing scarcity of CNTs in both the Ni and Co samples as metal concentration decreases and the decrease in intensity of the (002) graphite peak in Figure 3. The increase in D peak intensity for 200-1 and 2000-1 ratios is attributed to the increasing sp3 carbon content, which also contributes to sp2 radial breathing modes (RBMs).

**Figure 6:**
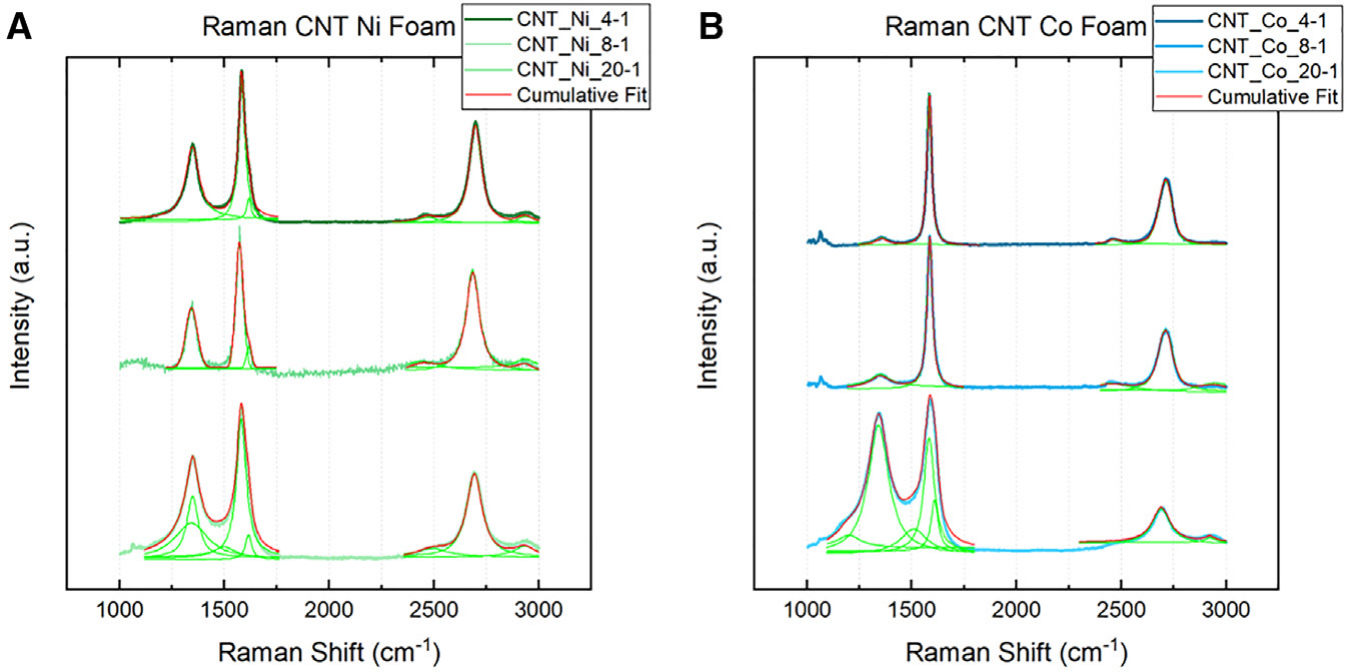
Raman peak analysis by Lorentzian fitting characterizes the electronic structure of the carbon materials synthesized using the 4-1, 8-1, and 20-1 ratios (A) Ni (B) Co.

**Figure 7:**
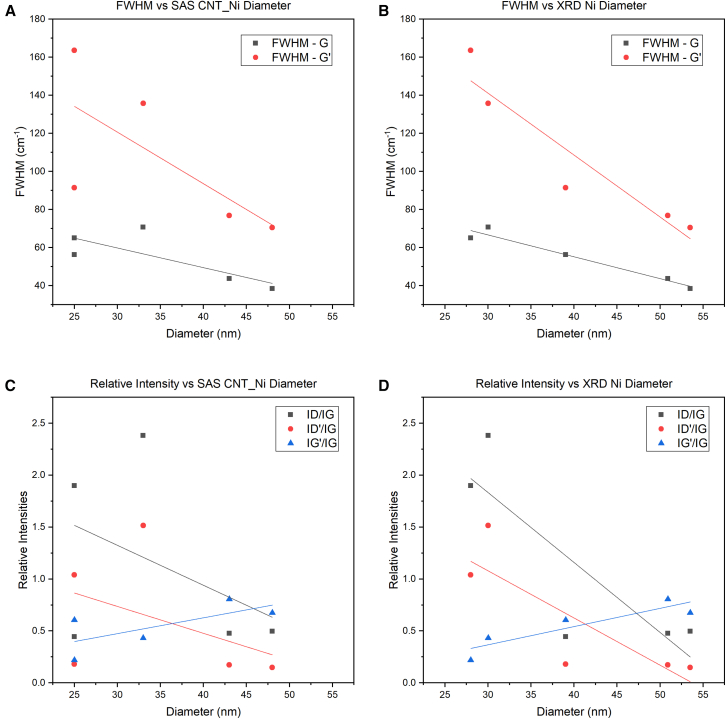
Comparison of CNT diameter calculated using SAS statistical software versus Ni grain size diameter calculated by Scherrer’s Eq. using FWHM of XRD Spectra in Figure 3 to investigate the scaling of Relative Intensities and FWHM with catalyst diameter (A) The plot of FWHM vs. SAS (B) The plot of FWHM vs. Scherrer Eq. (C) The Plot of Relative Intensities vs. SAS (D) The Plot of Relative Intensities vs. Scherrer Eq.

A closer look at the peak fitting for 4-1, 8-1, and 20-1 ratios for Ni and Co treated by CVD is shown in Figure 6. The presence of the u 1 and u 3 peaks are indicative of the relatively high ratio of carbon to metal still present at the 20-1 ratio for both Ni and Co. The appearance of the D′ peak signals the presence of MWCNTs in both Ni and Co 20-1 samples, as it does not appear in the Raman spectra of SWCNTs.^19^^,^^36^ However, the decrease in the relative intensity of the D peak to the G peak (ID/IG) may indicate the splitting of the D peak into D1 and D2 peaks in the Ni 20-1 spectrum, which emerge at graphite edges,^36^ where defects exist. Similar defects may be present in Ni 20-1 MWCNTs. A larger D is measured in the Co 20-1, indicating the potential for greater sp3 than Ni 20-1. This is also observed in the micrographs in Figure 2, where CNTs are generally shorter and sparser. Double resonance causes the 2D to be found in both Ni and Co 20-1 samples at approximately 2700cm^-1^,^19^^,^^37^ along with a peak at 2900cm^−1^ that can be associated with hydrogenation.^38^ An overtone of the power acoustic branch away from the K point leads to detecting the G^∗^ or D + D″ at 2400cm^−1^.^15^^,^^39^ At the 8-1 and 4-1 ratios, a significant difference is spotted in the Raman spectra between Ni and Co CVD-treated foams. In Ni 8-1 and 4-1, characteristic MWCNTs peaks such as the D, D′, and G peaks are detected. At smaller catalytic Ni particle diameters, the behavior of ID/IG, ID'/IG, and IG'/IG is known to scale with reciprocal diameter (1/d) and, therefore, the diameter of the catalytic nanoparticles. This was not observed in the Ni MWCNT in Figure 7; neither SAS nor Scherrer diameter produced a linear fit. This behavior may only hold for particles 20nm or less, as observed by Antunes et al.^19^ The behavior of the G and G′ FWHM was observed to fit linearly with both SAS and Scherrer estimated diameters, although Scherrer analysis measured better R^2^ fit than SAS. Raman spectra for the Co 4-1 and 8-1 ratios see a reduction in the D peak, suggesting less distortion in the electronic structure of the carbon. The *I*_*G'*_*/I*_*G*_ of 0.43 suggests that multi-layer graphene is present for 4-1 and 8-1 Co samples.^15^^,^^40^^,^^41^^,^^42^ The lack of a (002) graphite peaks in Figure 3B and as well as SEM micrographs seen in Figure 6D, agree with the notion that carbon enveloping the porous foam structure is multi-layer graphene, or at least an sp2 carbon lacking hexagonal crystal structure and AB stacking such as turbostratic graphite.^42^ In any case, MWCNTs are not observed in the Co 4-1 ratio and are rare in the 8-1 ratio.

**Figure 8:**
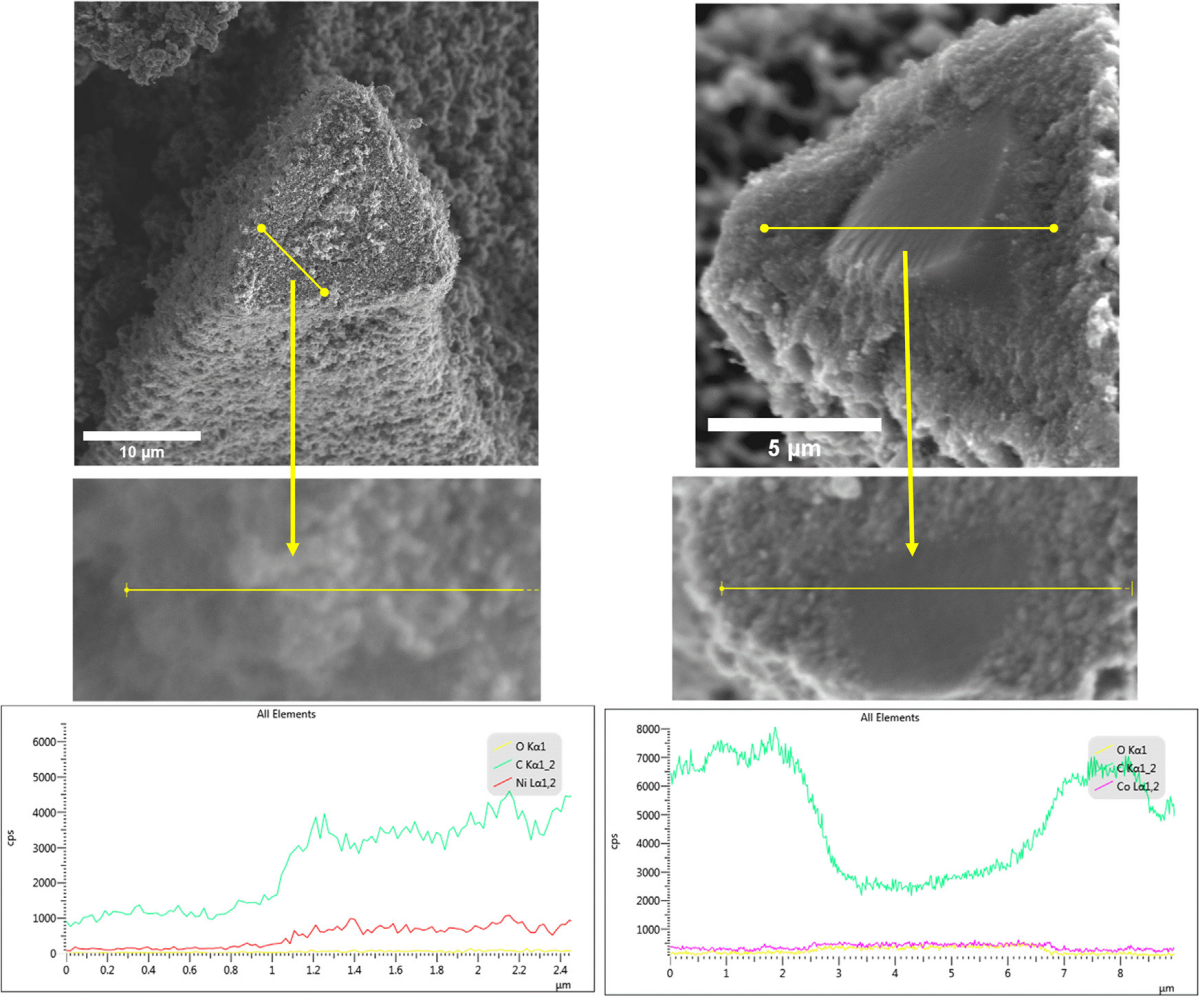
EDS line scans They were taken at exposed joints or struts in the CVD-treated carbon foams, with Ni on the left left (with a 10 μm scale bar) and Co on the right (with a 5 μm scale bar).

Carbon ‘plates’ with approximately 100-300nm diameter are pictured in Figure 6D. Estimation of Co grain size by Scherrer Eq. calculates diameters of 81.48nm and 54.99nm for 4-1 and 8-1, respectively. These grains may be close enough to form a substrate where the carbon phase is separated. To investigate the internal structure of the metal-carbon foam substrate, EDS line scans were performed on exposed cross-sections of carbon foam joints or struts, as seen in Figure 8. The line scans for the 4-1 Ni sample joint show a consistent presence of carbon and Ni. MWCNTs can be seen in the SEM micrograph as well. This suggests that the carbon solubility of Ni is not so low that the separation of Ni is so much so that clusters of Ni atoms during annealing agglomerate into particles greater than 54nm, as estimated by the XRD spectra for the Ni 4-1 ratio. The micrograph for the 8-1 Co sample treated with CVD synthesis exposes a core-shell structure of the foam joints. Line scans of the region show a decline in carbon content with a simultaneous rise in Co signal, though only slightly. This suggests the core is significantly lower in carbon content than the shell. In contrast with the Ni 4-1 sample, the carbon solubility of crystalline Co is lower (0.2–0.3%) than that of crystalline Ni (0.29%) (atomic % C) at the temperatures at which CVD synthesis takes place.^15^^,^^18^^,^^43^ Larger Co grains could be agglomerating into plates between 100 and 300nm in diameter, creating a substrate for graphene growth. Ni grains' slightly higher carbon solubility may be above the threshold for platelets to form, keeping their spherical shape and nucleating CNTs. The difference in crystalline carbon solubility may affect the diameter at which these nanoparticles and grain sizes nucleate.

Suppose we are to consider the MD models of F. Ding et al. In that case, we must consider a catalytic metal particle with a crystalline core and a shell that behaves as a liquid because of its greater atomic distances than allowable for a crystalline solid, as detailed by the Lindemann index.^44^ The carbon solubilities of crystalline metals must be balanced by the carbon solubility of liquid Ni and Co, which are an order of magnitude higher and reversed: Co having higher carbon solubility (2.68%) than Ni (1.97%) in the liquid state.^21^ The liquid behavior of the shell is due to two components: the carbon diffusion into the catalyst particle and the size effect. Because the interface between phases is defined as being at equilibrium, phase diagrams can give insight into the behavior of the metal particles during CVD. Larger Co particles (estimated grain size between 55 and 82nm by XRD for 4-1 and 8-1 ratios, respectively) have a higher crystallinity and, therefore, a larger fraction of carbon solubility between 0.2 and 0.3%. The liquid shell of these larger Co grains would also have a smaller fraction of the crystalline Co, thus having a smaller fraction of higher carbon (2.68%) solubility in the liquid state. In contrast, the smaller Ni particles (between 51 and 52nm in the 4-1 and 8-1 ratios, respectively) would have a larger fraction of liquid shell to crystalline cores. This means that a larger fraction of the catalytic Ni nanoparticle would have liquid behavior and, thus, higher carbon solubility than a larger Co particle. Differences in the solubility of carbon in both the crystalline and liquid phases may explain the differences in particle size and, therefore, the morphology of the graphite allotropes separated from solid solutions of carbon and metal at the nanoscale.

### CNT carbon foam for selective oil sorbent

**Figure 9:**
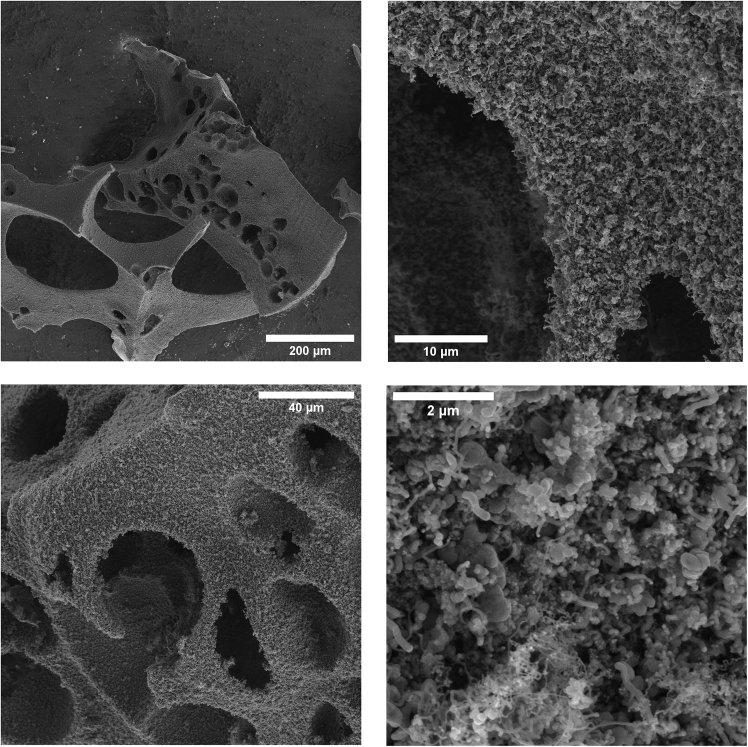
Hierarchical carbon foam is designed to retain oil capacity and repel water sorption All SEM micrographs are of the same sample, CNT@pC-4 at different magnifications (scale bars represent 200 μm, 10 μm, 40 μm, and 2 μm for top left, top right, bottom left and bottom right figures, respectively).

A hierarchical carbon foam was designed to provide enough capacity for oil sorption and synthesize MWCNTs on the surface of the foam to include hydrophobic properties in the foam. Hierarchical carbon foams were designed by annealing Ni carbon foam. However, when milled using mortar and pestle, the crushed powder was sieved between a 500 μm and 100 μm mesh. The carbon foam chunks milled to this specification were then exposed to CVD treatment for the growth of MWCNTs in the same conditions as earlier. Figure 9 shows the as-prepared hierarchical carbon foam at different magnifications. The same sugar-to-Ni ratios were chosen to test the effect of CNT growth on hydrophobicity. This size was chosen as small enough to maximize the surface area of hierarchical foams and large enough to retain oil.

**Figure 10:**
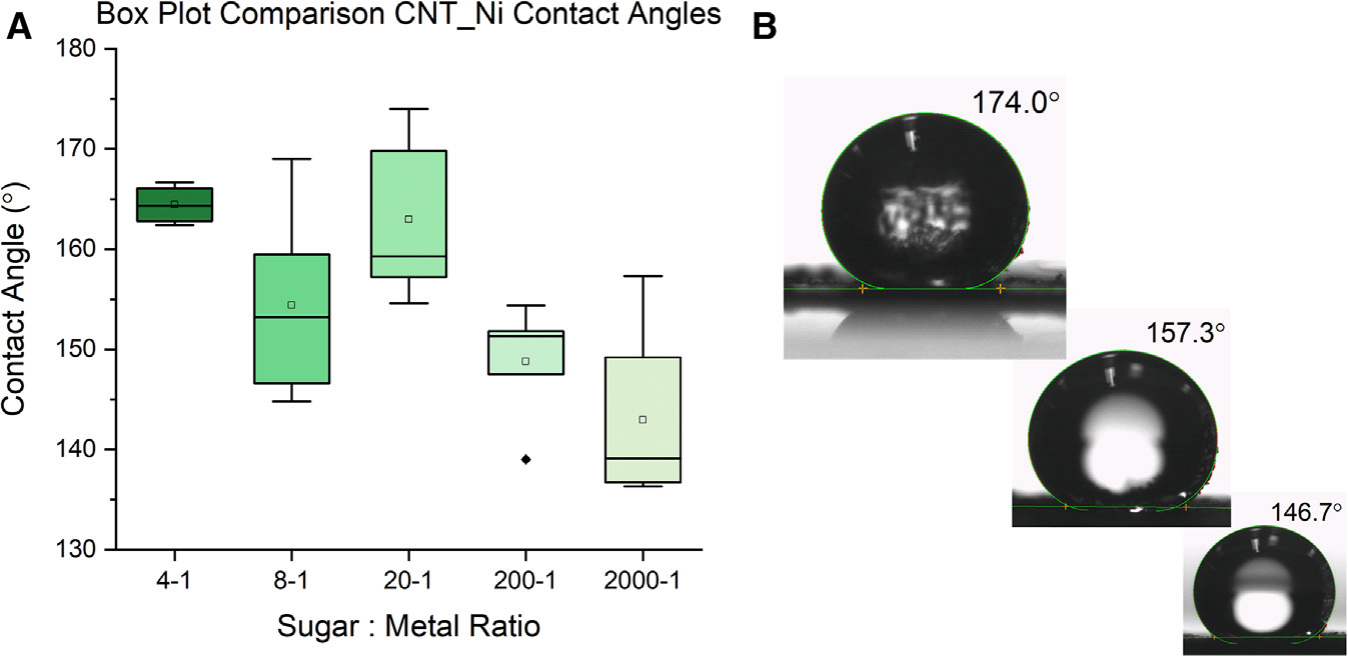
Boxplots (A) The distribution of contact angles measured for 4-1, 8-1, 20-1, 200-1, 2000-1 sugar to Ni ratios. (B) The variety of contact angles measured by goniometer.

CNT-coated carbon foams were used to coat a glass slide, and contact angles were measured using goniometry. Water was dropped in 10 μL increments onto coated slides, and angles were recorded using Sessile Drop fitting. Figure 10A shows boxplot comparisons of the contact angles measured for increasing ratio of Sugar - Ni. Low ratios of Sugar - Ni contain more metal content and produce CNTs with diameters ranging from 25nm to 48nm. Higher ratios of Sugar - Ni contain CNTs of similar tube diameter, but the frequency of CNT growth and availability significantly decreases. This is suggested in Figure 10A, where a negative trend follows the increase in the sugar-ni ratio. Contact angles for 4-1, 8-1, and 20-1 ratios demonstrate superhydrophobicity, displaying average contact angles of 164.5°, 154.4°, and 162.9° respectively, all of which exceed the definition of >150°.^45^

**Figure 11:**
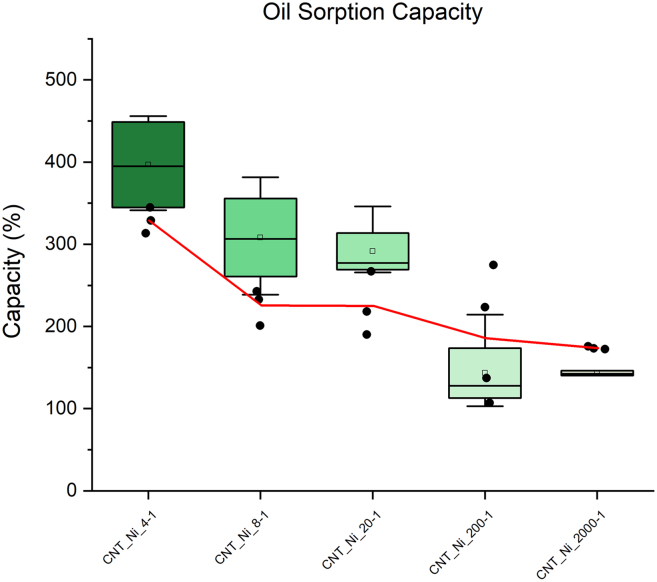
Oil sorption measurements of the hierarchical CNT carbon foam Boxplots of the measurements are presented. The red line denotes the average of the oil sorption for annealed foam chunks with particles before CVD treatment.

The oil sorption capacity for Ni-carbon foams treated with CVD synthesis was tested with a vacuum filtration set atop an Erlenmeyer ask with a vacuum nozzle. The filter paper was placed in the filter holder and saturated with oil. The mass of the oil-saturated filter paper was measured and subtracted from the mass with carbon foam particles and carbon foam particles saturated with oil. Figure 11 displays a boxplot chart of the mass percent the CVD-treated carbon foam can absorb. The red denotes a baseline oil sorption of carbon foams without CVD treatment. CNT@pC-Ni4 shows the highest oil sorption capacity, while CNT@pC-Ni200 and CNT@pC-Ni2000 exhibits much less capacity. As shown in Figure 10, the contact angles of CNT@pC-Ni200 and CNT@pC-Ni2000 is smaller than other sugar-metal-ratio samples, indicating less hydrophobicity. Therefore, less oil uptake is observed. In addition, as presented in Figure 4, the diameter of MWCNT grown on these two samples is smaller than the other samples, which could also result in less oil absorption due to less porosity and less pore size on the MWCNT structure.

It should be noted that the size of the sieved carbon foam is important to the oil capacity, and larger particles sieved in the 2 mm to 500 μm range would retain more oil. To maximize oil sorption with hydrophobicity, a systematic study to find an optimal carbon foam particle size is suggested.

### CNT carbon foam for hybrid supercapacitors

The abovementioned synthesis procedure was co-opted for synthesizing <20 m-sized carbon foam chunks with embedded Ni particles to grow MWCNTs. The pressure of the CVD process was increased from 100 torr to 250 torr to increase the length of MWCNTs, and the time was the same. Annealing pressure was the same at 100 torr during the flow of Ar and H_2_. The abundance of MWCNTs when using the 8-1 sugar to Ni ratio increases the surface area of the foam chunks and creates porosity within the MWCNT matrix.

**Figure 12:**
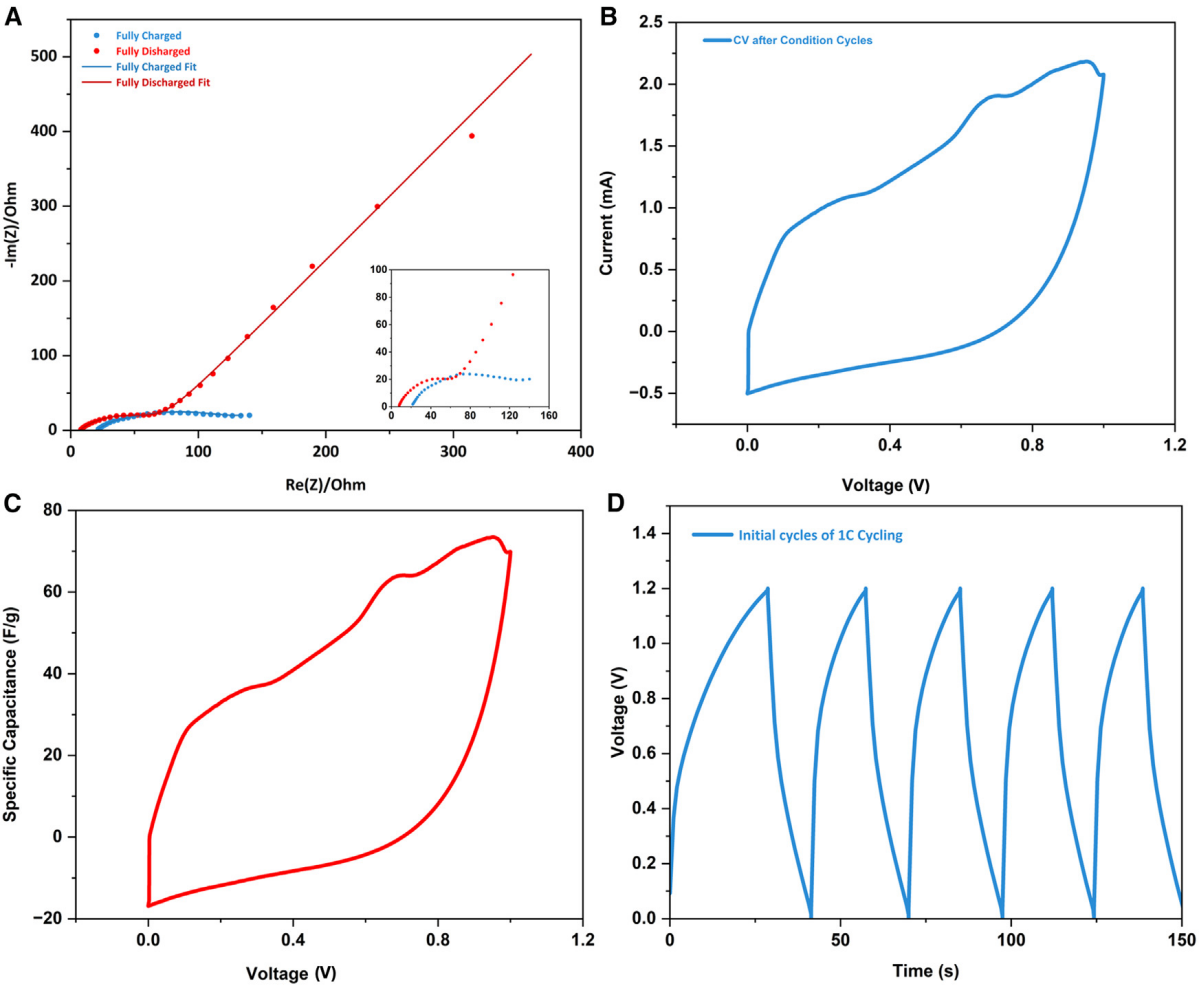
Electrochemical measurements of symmetrical aqueous cells (A) EIS data of the fully charged and fully discharged cells. (B) CV result at the scan rate of 5 mV/s. (C) Specific capacitance vs. voltage. Plot derived from the CV curve. (D) Galvanostatic charge and discharge profile of the symmetrical cells.

**Figure 13:**
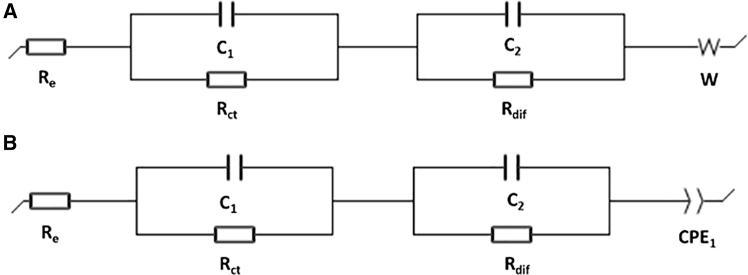
The equivalent circuit (EC) fitting from the EIS curve of symmetrical CNT cells (A) The EC at a fully discharged state. (B) The EC at a fully charged state.

Electrochemical double-layer capacitor (EDLC) electrodes were constructed using CNT foam as an active material to test the energy storage applications of the CNT foam. Symmetrical cells constructed with identical CNT active electrodes on each side with 6 M KOH as the electrolyte were first designed to study electrochemical performance. With the help of fast potassium-ion transfer in the aqueous electrolyte, the symmetrical cell exhibited low overall electrochemical impedance, as shown in Figure 12A. EIS results in the plot include the impedance curve of the cell in a fully charged state and fully discharged state. The fully charged state curve shows barely any tail, indicating K^+^ are immobilized at the surface of CNT in the electric double layers. From the equivalent circuit fitting as shown in Figure 13, the symmetrical cell’s impedance consists of electrolyte resistance R_e_, diffusive resistance R_dif_, charge transfer resistance R_ct_, EDLC capacitance at the local CNT surface C_1_, capacitance C_2_ at the current collector surface, Warburg’s impedance W, and constant phase element (CPE). SEI resistance can be expected to be much smaller than other resistance. The first point of the curve at the high-frequency region is the value of Re, representing the electrolyte resistance as other circuit elements become nearly zero at 100 kHz. R_e_ at the fully charged state (Figure 13B, R_e_ = 20.01 Ohm) increased slightly from the fully discharged state (Figure 13A, R_e_ = 7.219 Ohm). This is probably attributed to the deficient K^+^ concentration after the charging cycle, as the ions are electrostatically agglomerated at CNT surfaces. Similarly, R_ct_ becomes larger at the fully charged state (R_ct_ = 108.4 Ohm) than at the fully discharged state (R_ct_ = 60.94 Ohm) because of the static repulsion between the adjacent CNT surfaces as they are all surrounded by positively charged K^+^. R_dif_ also increased from nearly 0 when fully discharged to 5.386 Ohm as the cell was fully charged, owing to the complete intercalation of CNTs by K^+^. The straight lines sharply increasing at the low-frequency region of the discharged cell represents the Warburg’s impedance, which dominates capacitive behavior from the formation of ionic and electronic charges of the electric double layer (EDL) system at the CNT nanoporous surfaces; at this frequency, the ions can more easily diffuse into the nanoporous surfaces (Figure 6C). As the cell charges, the non-ideal capacitive behavior starts to form, which can be comprehended from the replacement of the Warburg element by CPE at a low-frequency tail.

CV test after the condition cycle of the symmetrical cell is presented in Figure 12B. Even though the CV curve shows a non-ideal rectangular shape, no sharp peaks were observed, indicating pseudo-capacitor behavior. The relationship between specific capacitance (F/g) and voltage (V) is plotted in Figure 12C based on the obtained CV data. Specific capacitance is calculated by the equation:

(Equation 1) $$C=\frac{I}{\Delta Vm}$$

where ΔV is the sweeping voltage step of the CV test, I is the current response of each voltage step, C is the specific capacitance at each voltage step, and m is the weight of porous carbon/CNT composite in the electrode. As the voltage sweep increased by 5 mV/s, the capacitance jumped quickly to 73.45 F/g, close to the specific capacitance of pure CNT-based electrodes.^46^

Figure 12D shows the galvanostatic charge and discharge profile in the initial cycles of the symmetrical cell. Smooth and straight lines are presented in both the charge and discharge process, showing the linear EDL charging and discharging behavior of the supercapacitor. The slope of the charging curve reduced after 0.7 V, which corresponds to the broad bump in the CV plot (Figure 12B). The slope change implies the initiation of a major diffusion process and intercalation of K+ on the interwall of CNT.^47^ The longer charging time than discharging time could be partially attributed to the incomplete K^+^ deintercalation in the first cycle and the formation of the SEI layer around the CNT.

**Figure 14:**
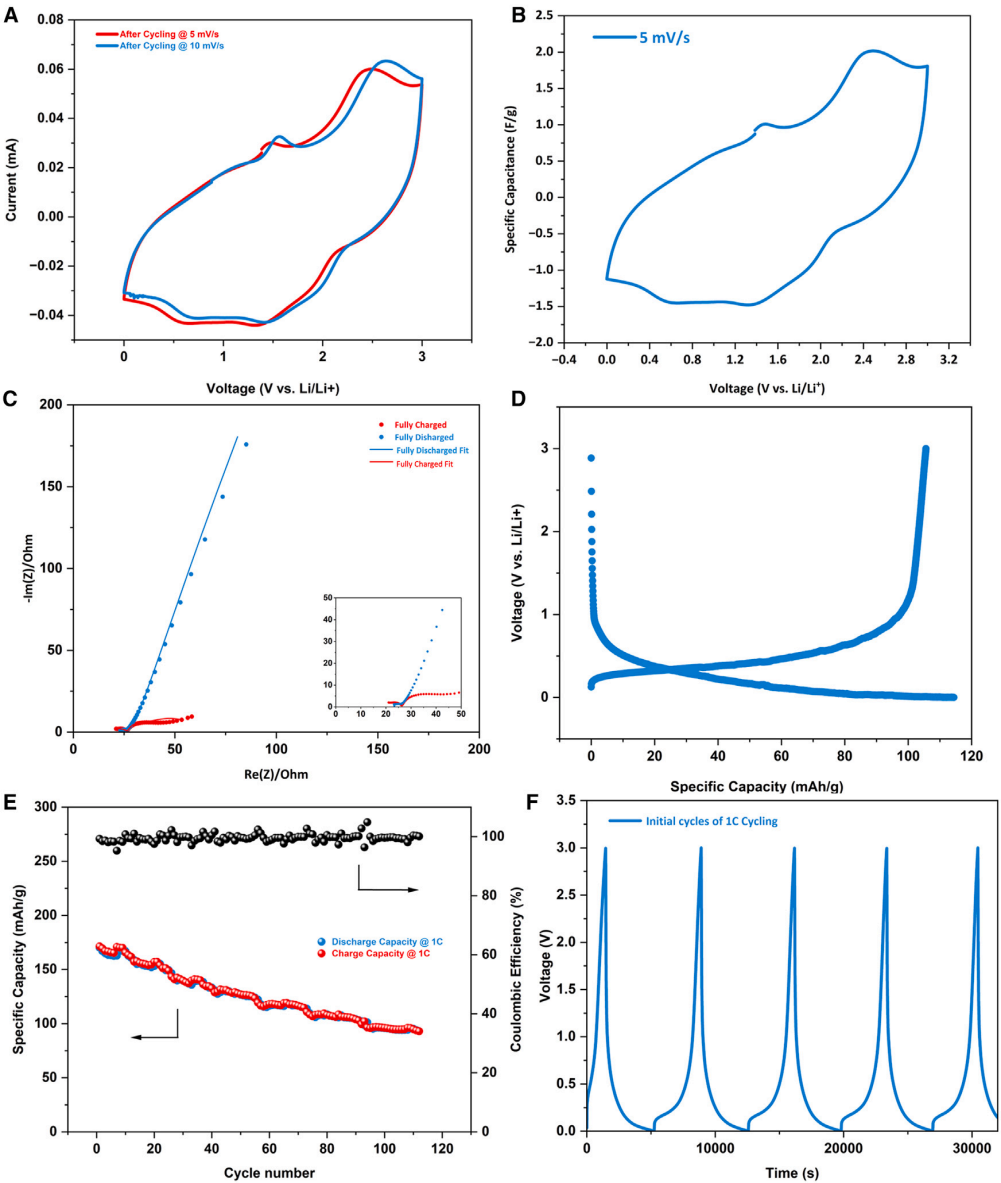
Electrochemical measurements of the hybrid supercapacitor (HSC) consisting of CNT cathode and Li metal anode (A) CV curves at 5 mV/s and 10 mV/s. (B) Specific capacitance obtained from the CV curve. (C) EIS result of CNT HSC. The red line shows the fully charged cell, while the blue line presents the fully discharged cell. (D) Voltage profile of the half cell at the 50th cycle. Wide plateaus of both the charging and discharging processes imply large diffusive capacitance in the electrode. (E) Cycling performance of the CNT HSC. (F) Galvanostatic charge and discharge profile of the CNT HSC.

**Figure 15:**
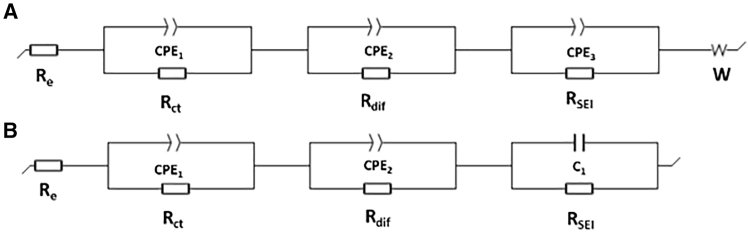
Equivalent circuit (EC) fitting from the EIS curve of CNT half cells (A) The EC at a fully discharged state. (B) The EC at a fully charged state.

The CNT foam was also fabricated into half-cell versus Li/Li^+^ to examine the capacitive and diffusive lithium storage capabilities. The resulting half-cell is identified as a hybrid supercapacitor (HSC) for its high capacity and high-rate capability. 1 M LiPF_6_ dissolved in EC/DEC (v/v = 1:1) was used as the electrolyte therefore the cation is Li^+^ and the anion is PF_6_^−^. The broad peak at 1.3–1.6 V in the CV curve (Figure 14A) represents lithium intercalation on the internal wall and external walls of the CNT.^47^ The calculated specific capacitance is shown in Figure 14B. The relative low capacitance compared to K^+^ symmetrical cell can be observed. Similar to the K^+^ symmetrical cell, low impedance was observed in the EIS plot of CNT half cells (Figure 14C). According to the equivalent circuit fitting (Figure 15) from the EIS data, R_e_ remained the same (18.59 Ohm charged vs. 18.97 Ohm discharged), indicating stable electrolyte resistance. Charge transfer resistance R_ct_ increased from 8.027 Ohm charged to 12.94 Ohm discharged, proving that the electrolyte-electrode ionic transfer process benefits from sufficient Li^+^ in the SEI layer at the charged state. Like K^+^ cells, the diffusive resistance R_dif_ decreased by 2–3 times in the Li^+^ half-cell from the charged state to the discharged state. SEI resistance is no longer negligible (R_SEI_ = 5.943 Ohm) at the charged state. In Figure 14D, the voltage profile of the CNT half-cell at the 50th cycle shows a wide and sloping plateau, which suggests that a diffusive process occurs from 0 V to 0.9 V (vs. Li/Li^+^). Therefore, combining the observation from CV, EIS, and voltage profile, a total pseudocapacity of 170 mAh/g in the CNT electrode is achieved with the help of both EDL and Li^+^ intercalation across the cycling voltage window. Furthermore, the cell capacity retained 55% after 110 cycles at 1C rate (Figure 14E). The capacity drop over cycling test is probably due to the cumulatively increased cell resistance, which is commonly observed in batteries.^48^^,^^49^ Figure 14F reveals the CNT half cells’ initial galvanostatic charge and discharge profile. The straight vertical line indicates EDL charge storage during the initial charging steps. After the curve enters the curving region, diffusive capacitance contributes to the overall capacitance.

Overall, the electrochemical measurements of the K + -based symmetrical cells and Li+-based half cells successfully proved that the hydrothermal-enabled porous carbon foams with embedded metal particles as substrates for multi-walled carbon nanotubes (MWCNTs) are functional and ideal for supercapacitor and battery applications. To further improve the electrochemical energy storage device performance, the following optimization needs to be considered: (1) complete carbon char removal, (2) Ni precursor removal after CNT growth, and (3) increased wettability of CNT with electrolyte by activation process.

### Conclusion

This study successfully demonstrates the utilization of porous carbon foam infused with metal nanoparticles, synthesized from basic polysaccharides and metal salts, as a foundation for cultivating nano-structured carbons in MWCNTs and multilayer graphene via CVD treatment. Integrating metal nanoparticles into carbon foams enhances the functionality of porous carbons and broadens their applications.

The saccharide-to-metal precursor ratio’s influence on the nano-structured carbon morphology was systematically investigated. SAS statistical analysis was employed to compare the diameters of carbon nanotubes at various saccharide-to-Ni ratios, with the results corroborated by XRD and Raman spectroscopy. When Co salts were substituted for Ni salt precursors, significant variations in carbon morphologies were observed and characterized using XRD, Raman spectroscopy, and EDS.

The resulting carbon nanotube-carbon foam composites were employed to fabricate hierarchical carbon foams. These foams demonstrate superhydrophobic and oleophilic properties, making them suitable for oil recovery applications. Additionally, these composites were utilized as active materials for supercapacitors and lithium-ion batteries. The supercapacitors exhibited a high specific capacity of 73.45 F/g when paired with K+ electrolyte and achieved stable cycling over 110 cycles when paired with Li+ electrolyte.

These findings highlight the potential of saccharide-derived porous carbon foams with embedded metal nanoparticles for advanced electrochemical applications, offering significant material functionality and performance improvements.

### Limitations of the study

The precise Ni/Co composition in the final carbon composites will be quantified in subsequent studies.

## Resource availability

### Lead contact

Requests for further information and resources should be directed to and will be fulfilled by the lead contact, Mihrimah Ozkan (mihri@ece.ucr.edu).

### Materials availability

This study did not generate new unique reagents.

### Data and code availability

• All the data generated in this study have been included in the article.
• This study did not generate a new code.
• Any additional information required to reanalyze the data reported in this paper is available from the lead contact upon request.

## Acknowledgments

The authors gratefully acknowledge financial support from Vantage Advanced Technologies LLC (award number 16040361), 10.13039/100007245Microelectronics Advanced Research Corporation (award number A003571404), International Chemical Systems, Inc. (award number 21020255), and the Office of the Vice-Chancellor for Research at the University of California, Riverside.

## Author contributions

M.O. and C.S.O. led the project, providing overall leadership and direction. Y.M. and W.L. designed and conducted all experiments, including material synthesis and all electrochemical tests. R.S. conducted material characterization, which included SEM, XRD, and Raman. F.V., P.P., A.P., E.J., and N.R. designed and conducted the oil sorption test and discussed all the experiments. All authors contributed to writing and refining the manuscript.

## Declaration of interests

The authors declare no conflict of interest.

## STAR★Methods

### Key resources table

**Table:** 

REAGENT or RESOURCE	SOURCE	IDENTIFIER
**Chemicals, peptides, and recombinant proteins**		

Ni (NO_3_)_2_ - Nickel (II) nitrate hexahydrate	Sigma-Aldrich	CAS No.: 13478-00-7
Co (NO_3_)_2_ - Cobalt (II) nitrate hexahydrate	Sigma-Aldrich	CAS No.: 10026-22-9
LiPF_6_ - Lithium hexafluorophosphate	Sigma-Aldrich	746711-100ML
Polyacrylonitrile (PAN)	Sigma-Aldrich	CAS No.: 25014-41-9
N-Methyl-2-pyrrolidone (NMP)	Sigma-Aldrich	CAS No.: 872-50-4

**Software and algorithms**		

EC-Lab software for Bio-Logic battery cycling and EIS (BCS810 and VMP3)	BioLogic	https://www.biologic.net/softwares/ec-lab-software/
ImageJ software 64-bit version	ImageJ	https://imagej.net/ij/

**Other**		

Bio-Logic battery cycling system BCS810	BioLogic	https://www.biologic.net/battery-cycler/
Bio-Logic electrochemical Impedance Spectroscopy (EIS) System VMP3	BioLogic	https://www.biologic.net/products/vmp3/
Raman Spectroscopy System LabRam HR800	Horiba	https://www.horiba.com/int/scientific/products/raman-imaging-and-spectrometers/
PANalytical Empyrean Series X-ray Diffractometer	Malvern Panalytical	https://www.malvernpanalytical.com/en/products/product-range/empyrean-range

### Method details

The author’s insights gained from examining the graphite encapsulation in carbon foams of Fe, Ni, and Co variants about the growth processes of CNTs on catalytic metal nanoparticles guided them toward the rational approach of trying to produce CNTs on the carbon foam that incorporates these three transition metals. The method described below factors in the temperatures required for graphitic encapsulation and the specific carbon solubility of Ni and Co. A comprehensive analysis of the carbon structures undergoing phase separation from catalytic metal substrates is provided, resulting in the formation of MWCNTs using Ni catalysts and multi-layer graphene using Co catalyst, given similar ratios of sugar to metal precursor.

#### Synthesis of Carbon Foam with Embedded Metal Particles

Carbon foams were created using methods akin to those previously employed by this group.^22^^,^^50^ The synthesis process involved the oxidation of a basic polysaccharide with metal nitrates and nitric acid, achieving a pH of 1 in a water-based solution. The xylose was selected as the sugar, while Ni(NO_3_)_2_ and Co(NO_3_)_2_ were chosen as the precursors for the metal nanoparticles. Ni and Co have sufficient carbon solubility to dissolve carbon feedstocks into a solid solution at elevated temperatures (627°C–950°C).^51^^,^^50^ The formation of eutectic compounds leads to phase separation^20^ and the precipitation of carbon allotropes such as graphite, graphene, and carbon nanotubes when the carbon content in the semi-liquid state of nano-sized particles increases. This is why Ni and Co were selected for incorporation into carbon foams. Sugar-metal molar ratios of 4-1, 8-1, 20-1, 200-1, and 2000-1 were used for comparison.

Graphite encapsulation within carbon foams has been observed and predicted to occur above 627°C.^18^ To prevent graphitization while encouraging the growth of catalytic metal nanoparticles, carbon foams were annealed at 600C for 1 h. Before the CVD synthesis of CNTs, annealed carbon foams were pulverized with a mortar and pestle to decrease the size of the particulate foam and sieved under 20 μm. This reduction in particle size increases the surface area and exposes more embedded metal nanoparticles to the gaseous carbon feedstock during CVD synthesis. To create hierarchical CNT foam for oil absorption and hydrophobic properties, annealed carbon foam pieces were ground until they were sieved between 500 μm and 100 μm mesh.

#### Growth of Carbon Nanotubes

The carbon foam, crushed to less than 20 μm, was positioned in a tube furnace to grow carbon nanotubes via CVD. The crushed carbon foam was subjected to an atmosphere with an equal ratio of H_2_ and Ar while the temperature gradually increased from 25°C to 750°C over 30 min. This process facilitates the reduction of any oxide layers coating the metal nanoparticles. The carbon foam was maintained at 750°C for an extra 30 min while the supply of H_2_ gas was turned off, and acetylene gas was introduced as the primary source of carbon in excess. The cooling down phase lasted for 45 min.

The resulting powder was then further hand-milled before entering the carbon etching process. The carbon char residue in the as-prepared carbon composite was partially removed by putting the sample under Ar/CO_2_ (both under 200 sccm) flow for 30 min at 500°C.

#### Electrode slurry preparation

The carbon composite containing porous carbon and CNT with xylose-to-Ni precursor ratio of 20:1 (CNT@pC-Ni20) was mixed with the conductive additive acetylene black and 5% polyacrylonitrile (PAN) in the NMP binder solution by a Thinky mixer (ARE-310, Thinky Inc.). The resulting slurry was evenly coated onto copper foil and fully dried in an oven at 80°C. The weight ratio of CNT@pC-20, AB, and PAN in the dried slurry is 85:7.5:7.5.

#### Coin cell assembly

Electrode disks were punched into 16 mm diameter. Coin cells were then made with different configurations for electrochemical tests. Half cells are made of VC active electrodes and Li metal as the counter electrode. 1 M LiPF6 in EC/DEC (v/v = 1:1) was used as the electrolyte for half cells. Symmetrical cells consist of CNT@pC-20 active electrodes on each side. The sample powder was soaked in concentrated KOH solution for activation before symmetrical cell assembly. Then the 6 M potassium hydroxide (KOH) aqueous solution was used for symmetrical cells.

#### Synthesis and procedure for selective oil sorbent CNT carbon foam

Hierarchical carbon foams were designed by annealing Ni carbon foam. However, when milled using mortar and pestle, the crushed powder was sieved between a 500μm and 100μm mesh. The carbon foam chunks milled to this specification were then exposed to CVD treatment for the growth of MWCNTs in the same conditions as earlier. The same sugar-to-Ni ratios were chosen to test the effect of CNT growth on hydrophobicity. This size was chosen as small enough to maximize the surface area of hierarchical foams and large enough to retain oil. The CNT@pC-Ni20 was selected for the compressor oil adsorption test. The oil adsorption capacity was calculated by the weight ratio between adsorbed oil and dry CNT@pCNi20 sample.

### Quantification and statistical analysis

A Bio-Logic battery testing system including modules BCS 810 and VMP 3 were utilized for all electrochemical evaluations. Before electrochemical tests, each cell underwent three conditioning cycles at 0.1 mA (C/10) to establish stable interfaces between the electrode and electrolyte. During the galvanostatic charge/discharge assessments of aqueous symmetrical cells, a constant current of 1 mA (1C) was maintained across 100 cycles within a voltage window of 0 V–1.2 V to avoid hydrogen evolution reaction (HER). For half cells, the galvanostatic charge/discharge voltage window was set to 0 V–3 V. Rate performance tests were carried out at 1C, 2.5C, and 5C for half cells. Cyclic voltammetry (CV) was conducted at voltage limits of 0 V–3 V versus Li/Li+ for half cells and −1.5 V–1.5 V for symmetrical cells, respectively. CV tests followed the 1st, 10th, and 100th galvanostatic cycles (1C), with scanning rates of 5 mV/s and 10 mV/s.

Electrochemical impedance spectroscopy (EIS) examined the cell’s internal resistance and polarization. EIS measurements were taken after fully charging and discharging the cells, with a perturbation amplitude of 10 mV and a frequency spectrum of 10 mHz–100 kHz.

### Additional resources

The as-prepared CNT foam and the CNT@pC-20 electrode’s surface morphology were examined using scanning electron microscopy (SEM, model NovaNanoSEM4 50), with a 30 kV accelerating voltage and a spot size setting of 3.5. The SEM chamber maintained a pressure of approximately 3 × 10^−3^ Pa, while the gun pressure and emission current were approximately 2.5 × 10^−7^ Pa and 75 μA, respectively. The electrode’s elemental composition was analyzed using energy dispersive X-ray spectroscopy (EDS, model NovaNanoSEM 450) and Raman spectroscopy (model Horiba, LabRam HR800), employing a 532 nm laser.

Crystal and molecular structure of the CNT foam samples were characterized and measured using a PANalytical Empyrean Series 2 Copper k⍺ source X-ray diffractometer. X-ray diffraction (XRD) readings were taken from 10 to 90° at a scan step size of 0.105 at 47.685 s per step.
